# Spatiotemporal dynamics of grassland aboveground biomass on the Qinghai-Tibet Plateau based on validated MODIS NDVI

**DOI:** 10.1038/s41598-017-04038-4

**Published:** 2017-06-23

**Authors:** Shiliang Liu, Fangyan Cheng, Shikui Dong, Haidi Zhao, Xiaoyun Hou, Xue Wu

**Affiliations:** State Key Laboratory of Water Environment Simulation, School of Environment, Beijing Normal University, Beijing, 100875 China

## Abstract

Spatiotemporal dynamics of aboveground biomass (AGB) is a fundamental problem for grassland environmental management on the Qinghai-Tibet Plateau (QTP). Moderate Resolution Imaging Spectroradiometer (MODIS) Normalized Difference Vegetation Index (NDVI) data can feasibly be used to estimate AGB at large scales, and their precise validation is necessary to utilize them effectively. In our study, the clip-harvest method was used at 64 plots in QTP grasslands to obtain actual AGB values, and a handheld hyperspectral spectrometer was used to calculate field-measured NDVI to validate MODIS NDVI. Based on the models between NDVI and AGB, AGB dynamics trends during 2000–2012 were analyzed. The results showed that the AGB in QTP grasslands increased during the study period, with 70% of the grasslands undergoing increases mainly in the Qinghai Province. Also, the meadow showed a larger increasing trend than steppe. Future AGB dynamic trends were also investigated using a combined analysis of the slope values and the Hurst exponent. The results showed high sustainability of AGB dynamics trends after the study period. Predictions indicate 60% of the steppe and meadow grasslands would continue to increase in AGB, while 25% of the grasslands would remain in degradation, with most of them distributing in Tibet.

## Introduction

The Qinghai-Tibet Plateau (QTP) is the largest geographical unit with the highest elevation on earth where grassland ecosystems dominate over 50% of whole plateau area^1^. It is also an important ecological and environmental area for water reservation, climate regulation and biodiversity conservation in the world and especially in Asia^2^. As an ecosystem that is highly sensitive to climate change, the grassland ecosystems of the QTP have increasingly become a popular research object for studying the global carbon cycle^3, 4^. Consequently, the aboveground biomass (AGB) in grassland ecosystems, which partially represents primary production, acts as a significant indicator of vegetation activity. Moreover, examining the spatial pattern of AGB dynamics can make contributions towards better understanding the responses of the grassland ecosystem to climate change, ultimately informing ecological regulation as well as regional policy-making^5–7^.

Traditional measurement of AGB by clipping and laboratory chemical analyses is destructive and expensive, and is constrained in terms of both temporal scale and spatial reach^8, 9^. With the development of remote sensing technology, satellite data with various temporal and spatial resolution are now widely used to study vegetation activities^10–13^. For instance, the normalized difference vegetation index (NDVI) is a vegetation index derived from reflectance in red and near-infrared wavebands, and has been demonstrated to be strongly correlated with grassland AGB in numerous studies^14–18^. The Moderate Resolution Imaging Spectro-radiometer (MODIS) NDVI products, which have high temporal and spatial resolution, have been applied in scientific research^19–21^. Furthermore, measurement of AGB using MODIS-NDVI products would overcome the shortcomings of traditional measurement, offering increased effectiveness.

Recent studies reported that some of the MODIS products, including LAI and fPAR, were not consistent with those derived from field measurements^22, 23^. Therefore, validation of the MODIS products is necessary to utilize these satellite products effectively. Such validation requires appropriate ground-based validation techniques or higher spatial resolution satellite data^24, 25^. Recently, portable spectrometers have been widely used for field measurements and are applicable for validating MODIS-NDVI data^24, 26^. After measuring the reflected radiation from plant canopies, NDVI data can be obtained using field portable spectrometers and remote sensing techniques^27, 28^. Spectrometers have a considerable advantage because they are not affected by atmospheric signal attenuation, and they measure the reflected radiation in fine spatial detail^29^.

Dynamic trend analysis of vegetation includes both the prevalent directions of vegetation dynamics in time-series during the study period, as well as possible directions of vegetation dynamics after the study period^30^. Among the methods that have been developed to explore the spatiotemporal dynamics of vegetation, linear regression analysis is the most widely used. The Hurst index, which calculates estimates through the R/S (Rescaled Range) analysis method, is mostly used to predict vegetation trends^31^. The Hurst exponent base method is useful for long time-series systems, even multi underlying activities systems, and can be better suited than correlation models to simulate future vegetation change under uncertainties in future climate changes. Although numerous studies have explored the relationship between NDVI and AGB in QTP grasslands and analyzed change trends of AGB, few studies have systematically analyzed spatial and temporal dynamic patterns of AGB in the QTP grasslands. Moreover, few studies have predicted future dynamic trends of AGB, and to date, the Hurst exponent has not been used for grassland AGB trend prediction in the QTP.

In this study, we investigated AGB and grassland canopy reflectance data from 64 plots in alpine steppe and meadow grasslands across the QTP during the growing season of 2013. High spatial resolution NDVI derived from a field spectrometer was used for validating the MODIS-NDVI data. The spatial and temporal patterns of grassland AGB of the QTP during 2000–2012 were then examined based on the established relationship between NDVI and AGB. Moreover, we also estimated the consistency of AGB dynamics after the study period. It is expected that understanding the variation characteristics and patterns of AGB in the QTP grasslands will promote ecological protection and management.

## Results

### MODIS-NDVI calibration

**Figure 1:**
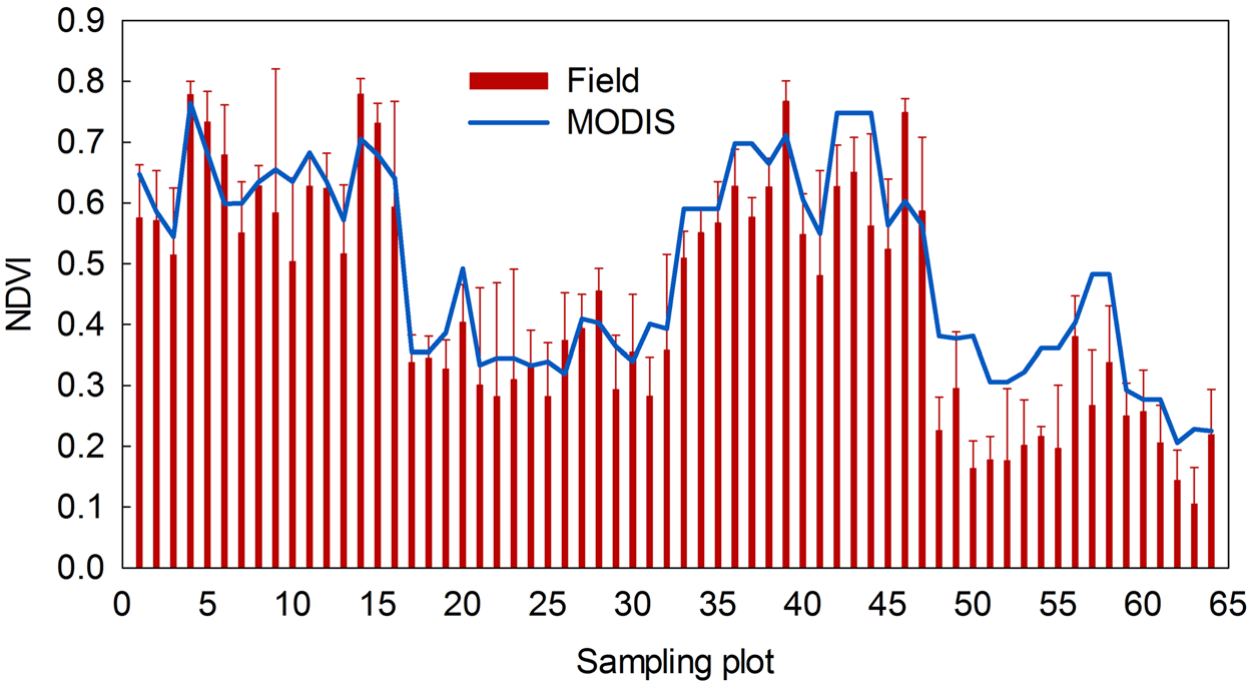
Comparison of NDVI calculated from MODIS and field-measured canopy reflectance data.

Plots 1–16 and 33–47 were in meadow grasslands, and plots 17–32 and 48–64 were in steppe grasslands. The NDVI values varied substantially across the plots because of differences in grassland density, ages, and types. Also, the values for the meadow plots were greater than for the steppe plots (Fig. 1). The NDVI values of the meadow plots, derived either from the MODIS or the field-measured reflectance data, were all greater than 0.5, while those for the steppe plots were less than 0.5.

**Figure 2:**
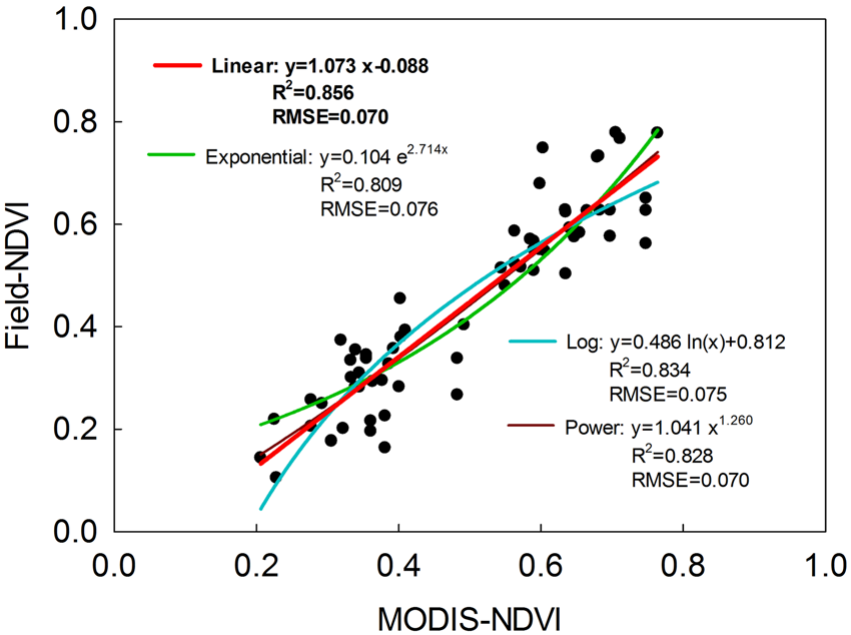
Regression models (linear, exponential, log, and power functions) of MODIS-NDVI and field-measured NDVI.

Although the MODIS-NDVI followed temporal patterns similar to the field-measured data, most of the NDVI calculated from field-measured canopy reflectance had smaller values compared with MODIS-NDVI at each sampling plot (Fig. 1). To calibrate the MODIS-NDVI data, four regression models (linear, exponential, log, and power functions) for each paired MODIS-NDVI and field-measured NDVI were estimated and compared (Fig. 2). The NDVI could be well simulated by these four regression models, and the best coefficient of determination (R^2^) and root mean square error (RMSE) were obtained while using linear regression, which indicated that the linear function performed best for calibrating MODIS-NDVI.

### Regression models of AGB and NDVI

**Figure 3:**
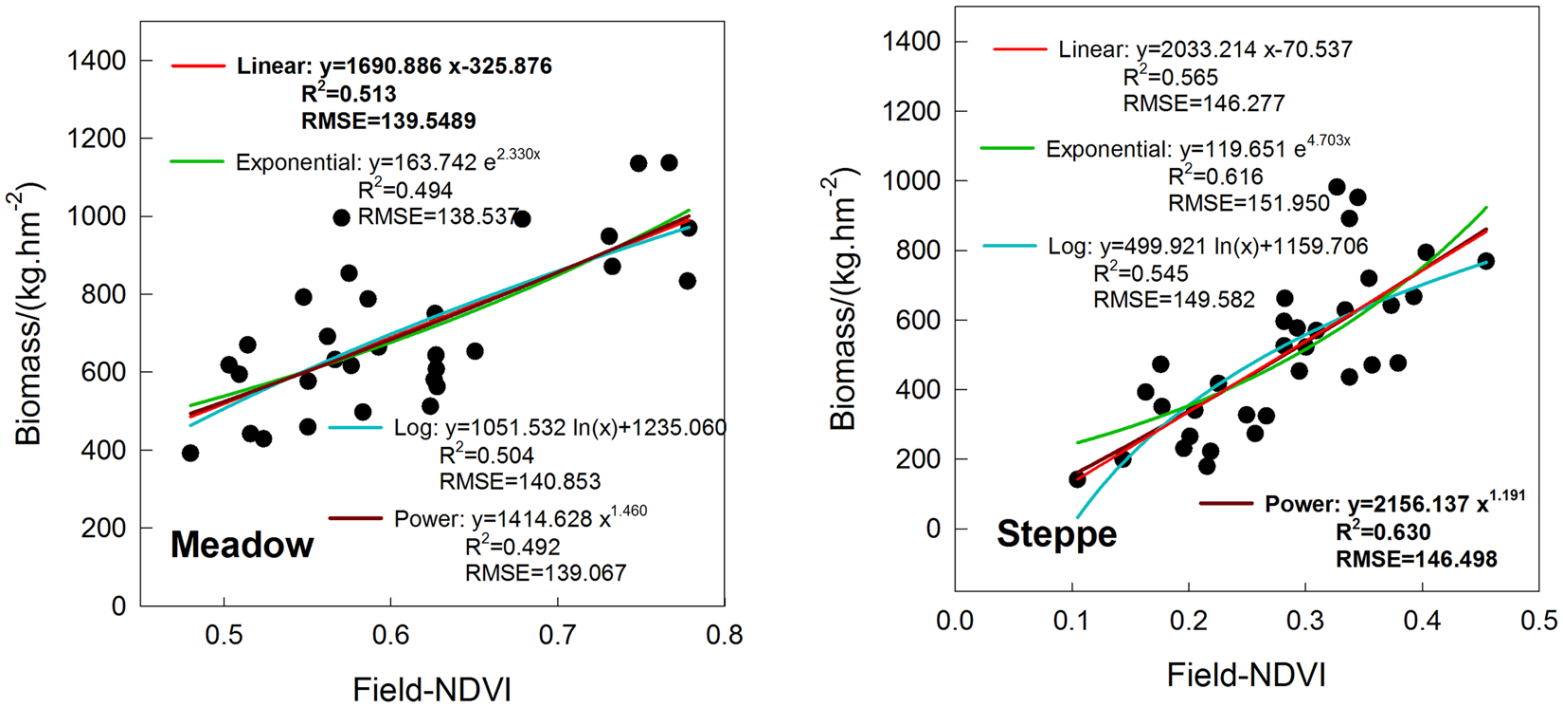
Regression models (linear, exponential, log, and power functions) of field recorded aboveground biomass and field-measured NDVI for the meadow and steppe.

The field-recorded AGB values of the meadow plots were relatively high, with an average AGB value of 706.7 kg/hm^2^, while, by comparison, the average AGB value of the steppe plots was 498.7 kg/hm^2^. The vegetation types, height, composition, and other properties are substantially different between the meadow and steppe, which could produce different AGB values even given the same NDVI values. The relationships between field-measured NDVI values and AGB were respectively explored for the meadow and steppe using the regression models (Fig. 3). Correlations between NDVI and AGB for the two grassland types were significant (p < 0.001) and met the assumptions for statistical analyses. For meadow grasslands, our analyses showed that a linear regression of yield as a function of AGB, expressed as NDVI, fits better than the exponential, log or power functions. The power function, with the R^2^ value of 0.63 and the RMSE of 146.498, was best suited for the estimation of the AGB in the steppe.

### Spatiotemporal patterns of AGB

**Figure 4:**
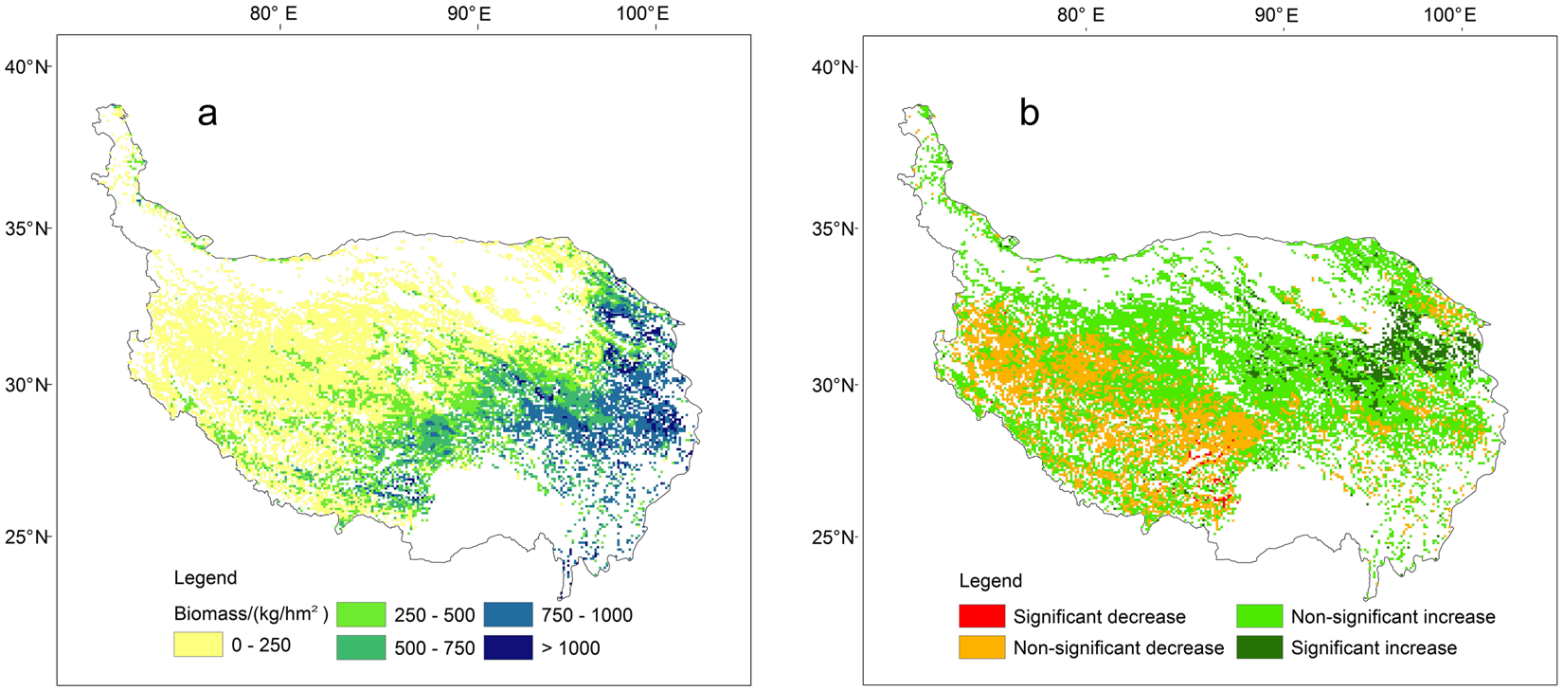
Spatial distribution of the mean aboveground biomass (**a**) and the change trend in estimated aboveground biomass (**b**) from 2000 to 2012 in the grasslands of the Qinghai-Tibet Plateau. The map was edited and generated with ArcGIS 10.2.2, http://www.esri.com/.

Using models established between NDVI and AGB, the averaged spatial distribution patterns of the grassland AGB in the QTP from 2000 to 2012 were estimated (Fig. 4a). The grassland AGB showed an increasing trend from northwest to southeast, and grasslands that had estimated AGB greater than 1000 kg/hm^2^ were mainly distributed in the eastern part of the QTP. Overall, more than 50% of the grassland estimated AGB was lower than 250 kg/hm^2^, and the average estimated AGB of meadow and steppe were 423.7 and 227.7 kg/hm^2^, respectively.

The AGB of the grasslands in the QTP increased during the study period, with slope values estimated from the linear regression analysis of the annual average AGB to be 0.315 and 0.134 for the meadow and steppe, respectively. This was also seen from the change trend analysis of AGB for all of the pixels (Fig. 4b). 70% of the grasslands experienced significant or non-significant increases in estimated AGB during the study period. Moreover, distinct spatial differentiation was observed in the change trends of estimated AGB. The grasslands that exhibited significant increases were mainly located in Qinghai Province, and the grasslands that exhibited significant or non-significant decreases were mainly located in Tibet.

### Sustainability of the AGB dynamic trend

**Figure 5:**
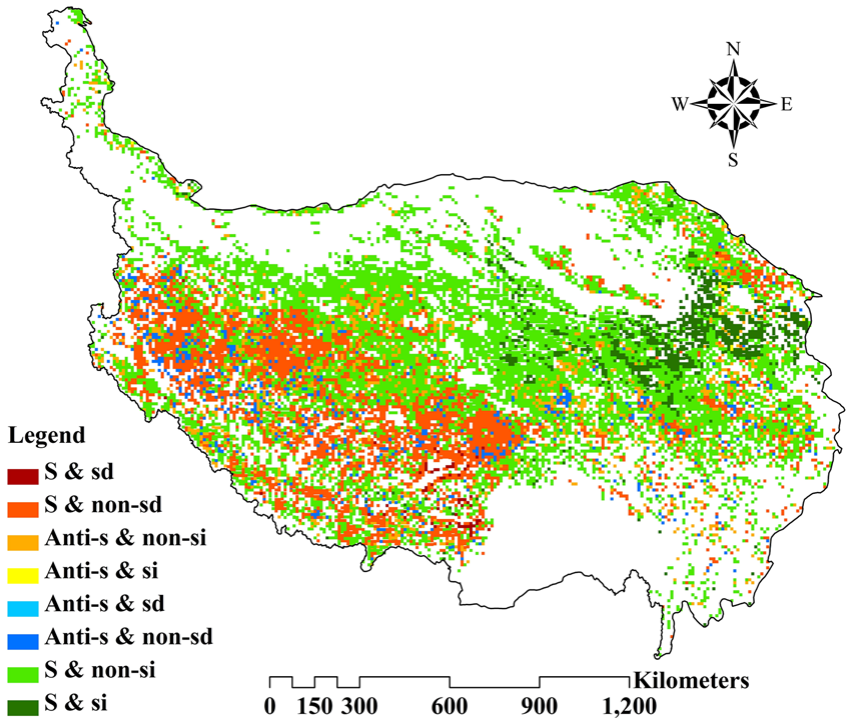
Spatial distribution of the AGB dynamics in the future based on trends during the study period and the Hurst exponent. S: sustainability; Anti-s: anti-sustainability; sd: significant decrease; non-sd: non-significant decrease; si: significant increase; non-si: non-significant increase. The map was edited and generated with ArcGIS 10.2.2, http://www.esri.com/.

Applying the R/S analysis to every pixel, the results showed that the regions with Hurst exponent greater than 0.5 accounted for 85.64% of the grasslands, which were primarily distributed in Qinghai Province and northern Tibet (Fig. 5). This indicates that the AGB dynamic trends of the grasslands were positively sustainable in the QTP. The average Hurst exponent value was 0.68 for steppe, and 0.66 for meadow, which meant that the steppe as a whole would have a higher sustainability level of AGB dynamic trends after the study period.

Coupled information of variation in trends and the sustainability of AGB combined analysis of slope and Hurst exponent (Fig. 5). The future AGB dynamic trends were uncertain when the grasslands showed anti-sustainability. The grasslands showing sustainability and improvement in AGB accounted for 60% of the total grassland, and those with sustainability and degradation accounted for 25%, which was mainly distributed in Tibet. Furthermore, 63% of the meadow and 58% of the steppe would consistently experience an upward trend in AGB values, while only 21% and 28% of the meadow and steppe would respectively show a downward trend.

## Discussion

### Application of field-measured spectrometer

Although remote sensing has been demonstrated to be an effective approach for characterizing AGB, few studies have examined the accuracy of the remote sensing data before using them. Some previous studies have explored the validation of MODIS products, including NDVI and GPP, using ground-measured spectra in Inner Mongolia and other regions of China^26, 32, 33^. However, few studies have focused on validating the MODIS-NDVI dataset in the QTP.

Ground measurements of grassland vegetation reflectance spectra are critical to evaluating uncertainties in MODIS-NDVI data that are derived from vegetation reflectance^32^. Lower quality MODIS-NDVI values were mainly due to the difficulties of “seeing” the surface because of frequent cloud cover^34^, and field-measured spectra overcome these difficulties. Moreover, the hyperspectral spectrometer used in this study has been demonstrated to be applicable for validating remote sensing data^24, 35^. In addition, the difference in scale between the field measurements and the remote sensing products will introduce some errors and biases into the validation^19, 25, 26^. To resolve this problem, we utilized a high-resolution MODIS-NDVI dataset and averaged the “point” measurements to represent the surface level in each vegetation plot under investigation, and the plot was designed to be as large as possible.

In our study, the field-measured NDVI and MODIS-NDVI data followed similar temporal patterns, which, to some extent, indicates the feasibility of our method. MODIS-NDVI is higher than field-measured NDVI from our findings is consistent with recent reports. Such difference is mainly due to the distinction in the sensor and band setting between our field (Equation 1) and MODIS-NDVI^36, 37^. MODIS data adopts the first two bands to calculate NDVI, and the bandwidths for the first and second band are 0.620–0.670 and 0.841–0.876, respectively. By contrast, in our calculation the bands were set at 0.680 and 0.800 for the red and near-infrared band.

Furthermore, the regression models established between the field-measured NDVI and AGB values in the steppe and meadow provide a new method for investigating AGB in QTP grasslands. Based on these regression models, the estimated AGB could be obtained simply using a handheld hyperspectral spectrometer in the field.

### Spatiotemporal patterns of AGB in the grasslands of QTP

Using regression models of the relationship between NDVI and AGB, spatiotemporal dynamics of AGB in QTP grasslands were investigated using validated MODIS-NDVI data during 2000–2012. The overall mean estimated AGB for the studied grasslands was relatively low (423.7 and 227.7 kg/hm^2^ for meadow and steppe, respectively). These estimated AGB values were smaller than those of some previous studies^38^, which may be due to the higher NDVI values those studies used to calculate AGB. The results from this study suggest an obvious increasing trend in estimated AGB from northwest to southeast in QTP grasslands. This heterogeneous pattern is mostly correlated with the distribution of different grassland types, which might be the result of differences in climatic factors and soil properties.

**Figure 6:**
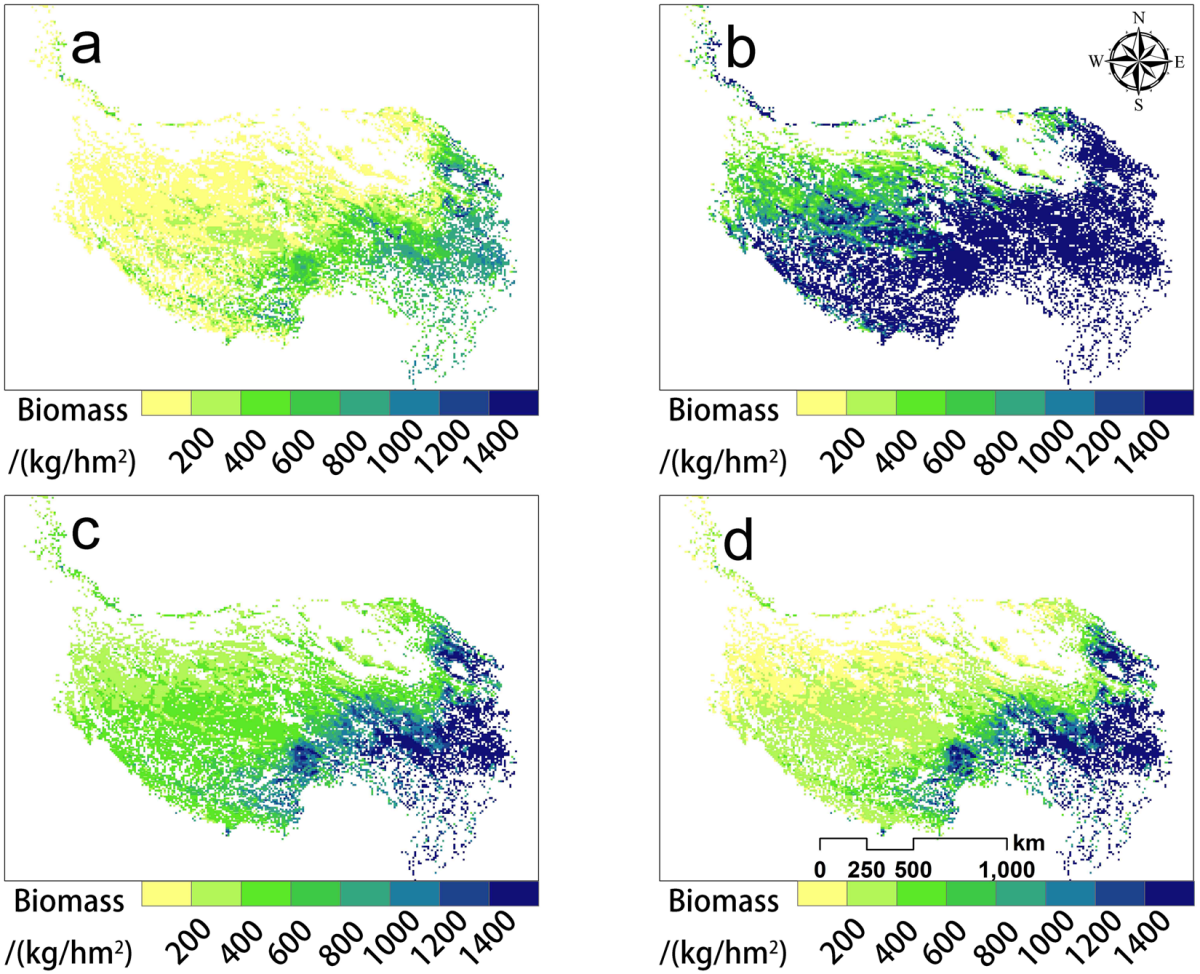
Spatial distribution of the mean aboveground biomass from 2000 to 2012 in the grasslands of the Qinghai-Tibet Plateau by different MODIS NDVI-AGB models. (**a**) Estimated by models of this study, (**b**) by Fang *et al*.^39^, (**c**) by Feng *et al*.^40^, and (**d**) by Chu *et al*.^41^. The map was edited and generated with ArcGIS 10.2.2, http://www.esri.com/.

Due to the different sampling strategies and the large estimated unit (10 km × 10 km) of AGB in this study, it is hard to directly compare our results with the field data in the past. Therefore, we chose the model of MODIS-NDVI and AGB constructed at the QTP in previous studies, and compared the predicted results determined by previous models alongside ours in order to evaluate the applicability of our models (Fig. 6). The spatial distribution of estimated AGB from different models was very similar, namely an increase in AGB from northwest to southeast. However, the value range of estimated AGB varied greatly among these models. The estimated AGB of Fang *et al*.^39^ and Feng *et al*.^40^ significantly overestimated the AGB of QTP grassland. In addition, Chu *et al*.^41^ also overestimated the AGB, given that the estimated AGB from Chu’s model reached as high as 2,378 kg/hm^2^ when the NDVI is 0.8. Our models are desirable in terms of the value range and spatial distribution, though there are still some shortcomings.

**Figure 7:**
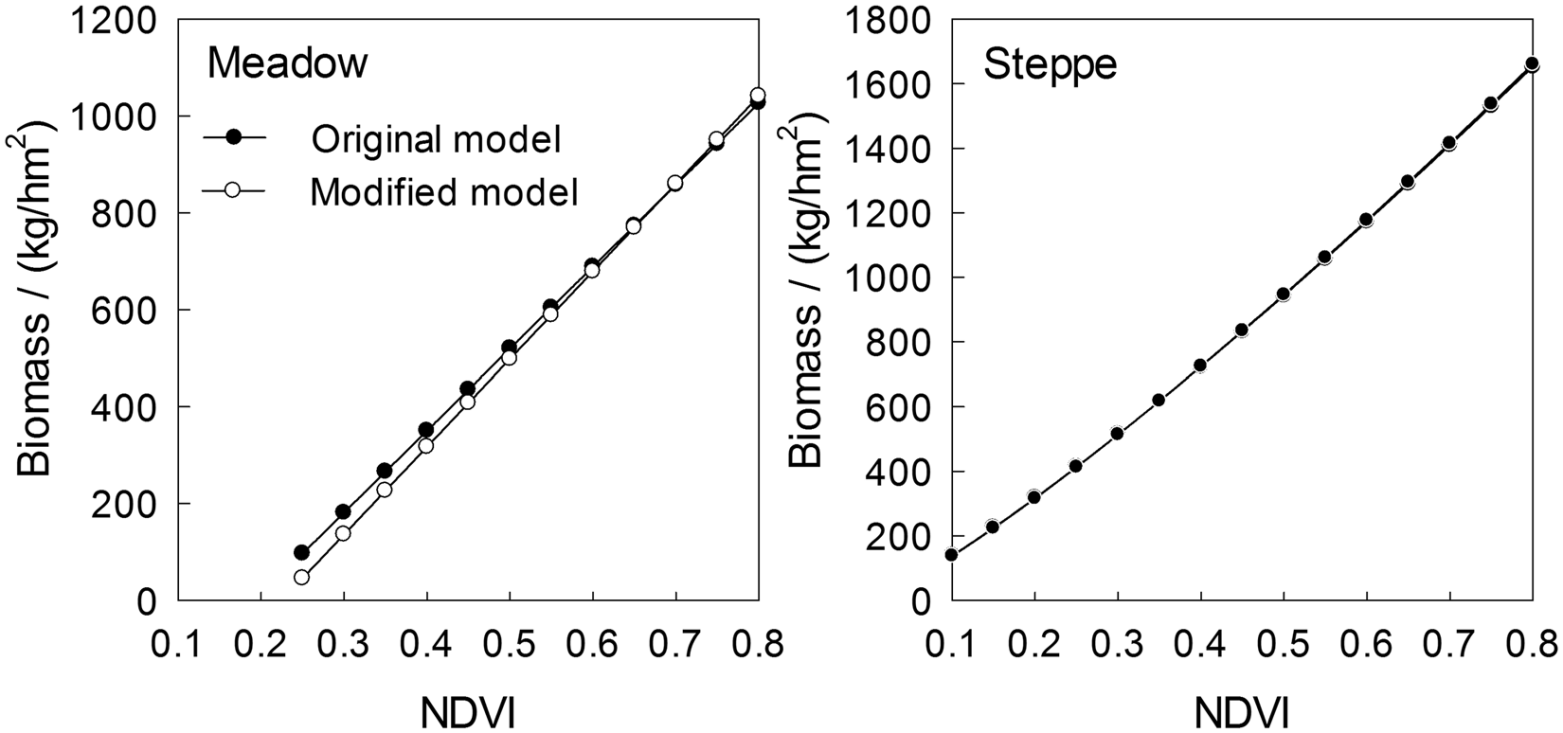
Comparison models based on all plots (original model) and selected plots (removed flower contaminated plots, modified model).

As mentioned in Methods section, both differences in the relationships between NDVI and AGB on the sub-vegetation type and flowers contamination will increase the uncertainty of our models^36, 42^. We did not discuss the potential uncertainty of estimated aboveground biomass due to sub-vegetation type in this study, but this will be addressed in following studies. Therefore, our discussion focused only on the impact of flower contamination. After removing severe flower-contaminated plots (coverage of large flower species above 20%), we rebuilt these NDVI and AGB models. Based on the rebuilt models, the effect of flower contamination is infinitesimally small for steppe, but it also indicates that our results might slightly overestimate AGB of meadow (Fig. 7). Therefore, further studies need to expand field samples to further quantify the effect of flower contamination on AGB estimation.

Linear regression analysis suggests an increasing trend of AGB during the study period. Previous studies that explored trends of grassland dynamics in the QTP using NDVI time-series also showed that the grasslands experienced upward trends in recent years^2, 43^. The primary factors influencing grassland dynamics are anthropogenic activities and climate change, and the grassland improvement is likely to be driven by the interaction of climate warming and human pressures^2, 44–46^. Distinct spatial differentiation of the change trends in estimated AGB was observed in Fig. 4b. The grasslands that showed decreasing trends were mainly distributed in the Tibet region, while the grasslands that experienced upward trends were mainly in the Qinghai Province. The spatial differentiation of the grassland AGB dynamics occurred as a result of the regional differences of climate change and human activities. For example, growing numbers of people and livestock in Tibet have caused grassland degradation, while a variety of ecological projects have been implemented in the Three-River Headwaters region of the Qinghai Province to restore and protect the grasslands^44, 47, 48^.

The combined analysis of the slope values and Hurst exponent has revealed the future tendency of AGB in the grasslands of QTP. As shown in Fig. 5, the Hurst exponent values for most of the grassland pixels were above 0.5, which indicated a high sustainability of AGB dynamic trends subsequent to the study period. More than half of the steppe and meadow grasslands would keep improving in AGB in the future. However, it is worth noting that 25% of the grasslands, mostly in Tibet, will continue to degrade. Therefore, appropriate measures and policies should be taken to prevent continuing grassland degradation in Tibet.

## Materials and Methods

### Study area

**Figure 8:**
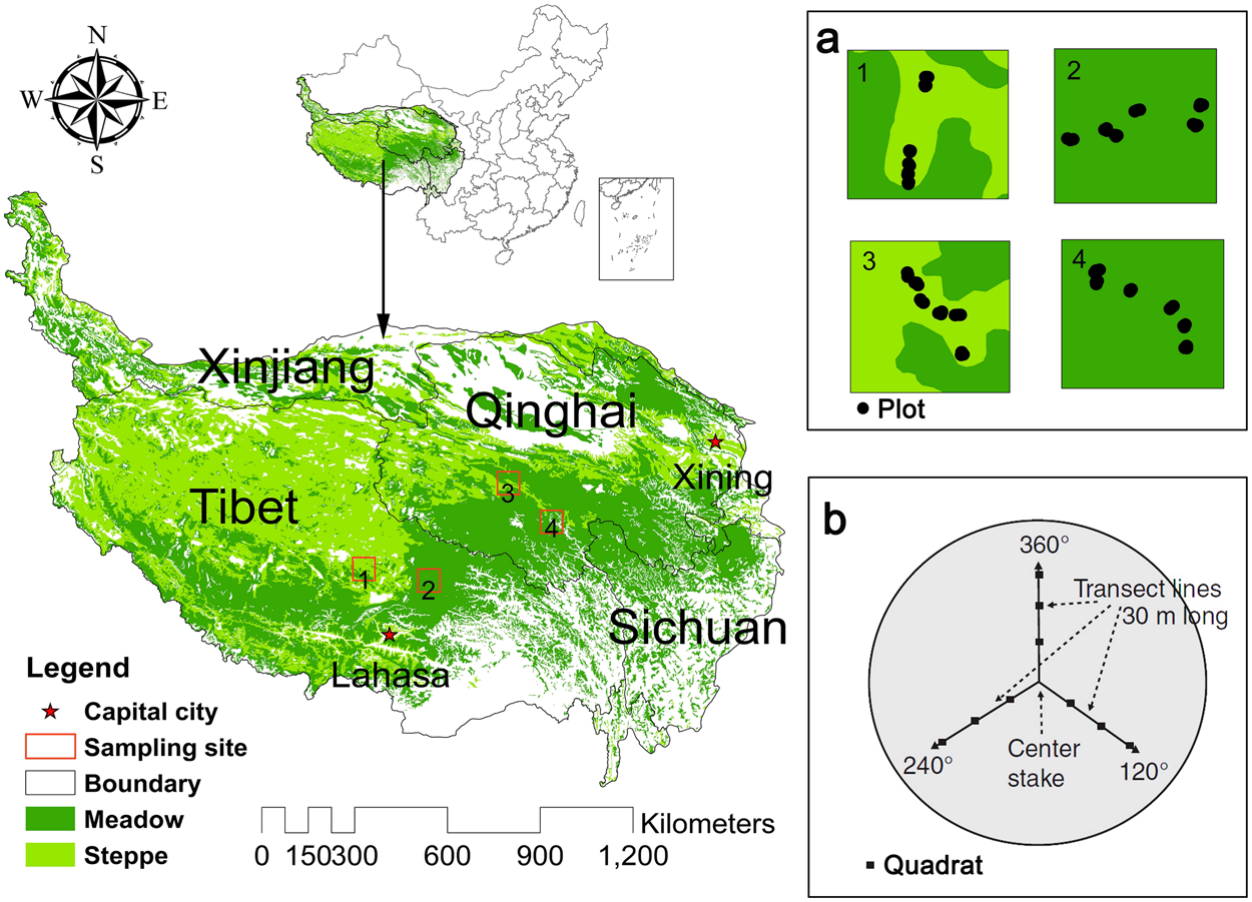
Location of the study area and distributions of the sampling sites. The words represent provinces in the Qinghai-Tibet Plateau. 1, 2, 3, and 4 represent sites of Baingoin County, Nagchu County, Quma River Township, and Qumarleb county, respectively. (**a**) Distributions of total 64 plots in four sites. (**b**) Distribution of 9 quadrats in each plot. The map was edited and generated with ArcGIS 10.2.2, http://www.esri.com/.

The Qinghai-Tibet Plateau (26°00′12″N–39°46′50″N, 73°18′52″E–104°46′59″E) is located in western China and covers the entirety of Tibet, Qinghai, and parts of Xinjiang, Gansu, Sichuan, and Yunnan Provinces in China (Fig. 8). It is the highest and largest plateau on earth, as the total area of the QTP is approximately 2.61 × 10^6^ km^2^ and the average elevation is over 4 km. The complex terrain and variable boundary conditions of the QTP create unique weather and climatic characteristics, which are cold and arid in the winter, and warm and moist in the summer^49, 50^. Grasslands, mainly composed of meadows and steppes, are the dominant vegetation type across much of the QTP. With the climate varying from cold-dry in the northwest to warm-wet in the southeast, the grassland types vary from steppe in the northwest to meadow in the southeast (Fig. 8).

## Experimental Design

A field survey was carried out in July and August in 2013 when the grasslands had peak biomass. According to vegetation map, topographic map, and road distribution maps of the QTP, we selected four sampling sites with the principle of reachability and typicality. In our field survey, we adopted a comprehensive sampling method to acquire more accurate values of AGB; in total, 576 small quadrats were included in these 64 plots (Fig. 8). This sampling method was recommended by Hankins *et al*.^51^ in 2005 for grassland survey rather than brief full coverage sampling. In fact, the distribution of our plots in every site was not very close—at least 1 km, approximately—and we also considered the spatial variation of different grassland types. In addition, at each plot, we ensured that the landscape was homogeneous within the corresponding pixel of the MODIS-NDVI product. There are many “no man’s lands” distributed in the western region of the QTP, including Altun National Nature Reserve, Changtang Nature Reserve, Hoh Xil Nature Reserve, etc. These regions were inaccessible and had high elevation and limited trafficability of land surfaces. Besides, the operational condition of hyperspectral spectrometer is strict, requiring sunny and cloudless weather.

Eventually, thirty-one plots were recorded in Qumarleb County and Nagchu County, where the grassland type was meadow, and 33 plots were recorded in Quma River Township and Baingoin County, where the grassland type was steppe. Such sampling sites were also the representative sampling areas in some other studies. Conventionally, the dominant plant species in the QTP grasslands are sedges and grasses, including *Poa crymophila* and *Kobresia humilis* in the meadow, and *Stipa purpurea* in the steppe. In our sampling sites, the identified dominant plant species conformed to these conventions. Moreover, sampling plots were randomly selected from the geographic map in each site. The sampling plots were enlarged in Fig. 8a.

At each field sampling plot, we randomly selected a circle with a 30 m radius, and the vegetation was sampled using the methods put forward by Hankins *et al*.^51^. Three transects with nine 1 m × 1 m quadrats were placed in each circle, and each quadrat was divided into a 10 × 10 grid with 100 intersecting points to investigate the vegetation types, heights, frequency, and coverage. We obtained vegetation biomass data using the clip-harvest method in the quarters of each quadrat^52^, and the total biomass was weighed after drying at 60 °C to a constant weight. At each sampling plot, the average biomass value of the nine quadrats was used to represent the grassland biomass.

Grassland canopy reflectance data were collected under clear sky conditions using a handheld, single-channel hyperspectral spectrometer (UniSpec-SC, PP Systems, Amesbury Mass, USA). One point was randomly selected in each quadrat to measure the canopy reflectance. The ground resolution for the canopy reflectance measurement was approximately 0.39 m^2^, depending on the height of the probe. The spectral data were processed to reflectance in the 400–1,000 nm range using MultiSpec software with a spectral resolution of 1 nm^35^.

### NDVI data

The MODIS-NDVI dataset used in this study was acquired from the National Aeronautics and Space Administration (available at http://daac.gsfc.nasa.gov/). Data from 2000 to 2012 with a spatial resolution of 1 km × 1 km and a temporal resolution of 16 days were used for the dynamic change analysis. To obtain the NDVI values that represented the period with the greatest abundance of vegetation within a year, the maximum value composite method was used to compose the annual NDVI of each raster^53, 54^. The value of the maximum compiled annual NDVI also eliminated the negative influence of seasonal changes in vegetation cover in different areas^55^. The annual NDVI data from 2000 to 2012 with 1 km × 1 km spatial resolution was reset to 10 km × 10 km using an aggregate method in ArcGIS toolbox.

The MODIS-NDVI data from July to August in 2013, when the survey was conducted, were compared with the measured data. The spatial resolution of the MODIS-NDVI data was different from that used for the dynamic change analysis, which was 250 m × 250 m. The MODIS pixels at the locations of the sampling plots were extracted separately for analysis. The measured NDVI was calculated from the reflectance using the following formula:

1 $$NDVI=\frac{({R}_{800}-{R}_{680})}{({R}_{800}+{R}_{680})}$$

where R_800_ and R_680_ are the reflectances at 800 nm and 680 nm, respectively. The wavelength selection was based on previous studies that used 680 nm as the red waveband and 800 nm as the near infrared waveband to calculate a narrow-band NDVI^24, 35, 56^. The average NDVI values of the nine quadrats were used to represent the NDVI value at each plot.

### Analysis of dynamics of aboveground biomass

In this study, regression models constructed by MODIS-NDVI and corresponding field-measured NDVI was used to calibrate MODIS-NDVI data during the period 2000 to 2012. We also regressed plot-specific AGB against corresponding field-measured NDVI data and established the relationship between the AGB and the calibrated MODIS-NDVI to estimate AGB at each pixel. NDVI could saturate at high vegetation density (typically with LAI higher than 3.0 for grassland)^57–59^. That is, NDVI increases little at LAI higher than 3.0 regardless of the further increase of LAI or biomass^37^. In our study, we did not consider the saturation condition of LAI in the AGB calculation as a whole, as there were no plots with NDVI larger than 0.8 in our field survey. Also, considering the LAI for most areas of Qinghai-Tibet plateau is less than 3 even in the growing season^60^, the AGB calculation in general is reasonable.

The relationship between NDVI and AGB depends on the vegetation type or sub-vegetation type^42^. However, through the field survey and the analysis of all species in each plot, we found that the dominant species of most plots are mainly *Gramineae* and *Cyperaceaem* families. In total, we analyzed that the important values of species belonging to these two families ranked first in 78% of the total plots. Besides, for some degraded alpine meadow, the NDVI could also be contaminated by flowers that are on the top of canopy^36^, affecting the relationship between AGB and NDVI. However, most samples were dominated by *Gramineae* and *Cyperaceae* families, and only four plots showed coverage of large flower species (i.e., *Polygonum viviparum*, *Potentilla chinensis*) above 30%. Therefore, flower contamination is not considered to explain our findings.

Linear regression analysis, which is simple and robust, is widely used for estimating vegetation change^61^. This method can simulate the vegetation change of each grid, thereby enabling the spatial characteristics of vegetation change to be determined^55^. In this method, time is the independent variable, and the AGB value of each pixel is the dependent variable; the slope of the linear regression of each pixel then is used to indicate the trend of AGB change across the period of interest^30^:

2 $$slope=\frac{n\times \sum _{i=1}^{n}i\times AG{B}_{i}-\sum _{i=1}^{n}i\sum _{i=1}^{n}AG{B}_{i}}{n\times \sum _{i=1}^{n}{i}^{2}-{(\sum _{i=1}^{n}i)}^{2}}$$

where n is the number of study years (13 in this study), *i* is the serial number of the year, and *AGB*_*i*_ is the AGB for year *i*. A positive grid slope corresponds to an increasing trend in AGB change over the 13 years, and a negative value corresponds to a decreasing trend in AGB change. To evaluate AGB change qualitatively, the slope values of all of the grids were classified into four levels: significant decrease (slope < −10), non-significant decrease (−10 ≤ slope < 0), non-significant increase (0 ≤ slope < 10) and significant increase (slope ≥ 10).

The Hurst exponent represents the changing trend based on previous long and positive time-series. For example, when the vegetation has a longer history of increasing AGB, the AGB status is more likely to be positively sustainable. Theoretically, the Hurst exponent is not affected by the magnitude of fluctuations, but by the sequential ordering of the fluctuations^62^. Therefore, this approach based on the Hurst exponent is extremely useful when a wide range of underlying activities exist within a system over a measured length of time^63^. Therefore, the prediction of Hurst exponent is based on the historical AGB conditions, and the prediction from Hurst exponent can well explain the trend of AGB under climate change and human activities. The Hurst exponent was calculated using the R/S analysis procedure: H is the Hurst exponent, which determines the randomness of the time series, and is obtained by fitting the equation log(R/S)_τ_ = a + H × log(τ) by the least squares method. When the Hurst exponent is equal to 0.5, the time series is random and without sustainability. When the value is closer to 0.5, the time series is more strongly random. When the value is greater than 0.5, the change trend of the future time-series is consistent with that in the study period, and a larger value means more sustainability. On the other hand, when the value is less than 0.5, the time-series will experience an opposite trend in the future, and smaller values indicate less sustainability over time.
